# Health Effects and Life Stage Sensitivities in Zebrafish Exposed to an Estrogenic Wastewater Treatment Works Effluent

**DOI:** 10.3389/fendo.2021.666656

**Published:** 2021-04-30

**Authors:** Ruth Cooper, Arthur David, Anke Lange, Charles R. Tyler

**Affiliations:** ^1^ College of Life & Environmental Sciences, University of Exeter, Biosciences, Exeter, United Kingdom; ^2^ School of Life Sciences, University of Sussex, Brighton, United Kingdom

**Keywords:** estrogen, effluent, chronic, development, zebrafish, transgenic, vitellogenin (vtg)

## Abstract

A wide range of health effects in fish have been reported for exposure to wastewater treatment work (WwTW) effluents including feminized responses in males. Most of these exposure studies, however, have assessed acute health effects and chronic exposure effects are less well established. Using an Estrogen Responsive Element-Green Fluorescent Protein (ERE-GFP)-Casper transgenic zebrafish, we investigated chronic health effects and life stage sensitivities for exposure to an estrogenic WwTW effluent and the synthetic estrogen 17α-ethinylestradiol (EE2). Exposure to the WwTW effluent (at full strength;100%) and to 10 ng/L (nominal) EE2 delayed testis maturation in male fish but accelerated ovary development in females. Exposure to 50% and 100% effluent, and to 10 ng/L EE2, also resulted in skewed sex ratios in favor of females. Differing patterns of green fluorescent protein (GFP) expression, in terms of target tissues and developmental life stages occurred in the ERE-GFP- zebrafish chronically exposed to 100% effluent and reflected the estrogenic content of the effluent. *gfp* and vitellogenin (*vtg*) mRNA induction were positively correlated with measured levels of steroidal estrogens in the effluent throughout the study. Our findings illustrate the importance of a fish’s developmental stage for estrogen exposure effects and demonstrate the utility of the ERE-GFP zebrafish for integrative health analysis for exposure to estrogenic chemical mixtures.

## Introduction

It is now widely accepted that various chemicals entering the environment via wastewater treatment works (WwTW) effluents can have endocrine disrupting effects in aquatic organisms, and notably in fish. Endocrine-related effects in fish associated with WwTW exposure include the induction of vitellogenin (VTG; an estrogen dependent yolk precursor), intersexuality (1–4), alterations in blood hormone levels (5–7) and impaired sexual development and reproduction (8–10).

Evidence from both *in vivo* and *in vitro* studies suggests that both endogenous and synthetic estrogens, including estrone (E1), 17β-estradiol (E2) and 17α-ethinylestradiol (EE2) are responsible for the majority of estrogenic activity observed in wastewater effluents and their receiving waters (11–13) but in some cases estrogen-mimicking chemicals, such as 4-nonylphenol (NP) and bisphenol-A (BPA) can also contribute to this activity, and collectively they are responsible for the WwTW exposure effects on reproductive development and function in fish (14, 15). Exposures to estrogenic chemicals in mammals have also been linked with wider health effects on the immune system, obesity and cardiovascular disease (16–18).

In the UK it has been reported that around one third of rivers in England may contain levels of environmental estrogens sufficient to cause disruption to reproduction in fish (3). This is highly variable however, and estrogenic discharges into these rivers from WwTWs is dependent on a variety of factors, including the type of wastewater treatment process and the operational conditions in those WwTWs (19), and the level of effluent dilution in the river. Seasonal factors can also impact on rates of chemical biodegradation (20) affecting exposure levels over time.

Although concentrations of estrogenic chemicals typically found in WwTW effluents and surface waters are generally low (in the ng to μg/L range depending on the specific chemical) many have been shown to bio-concentrate in aquatic organisms (21–23) and this may increase their biological effects over time [as evidenced by (24, 25)]. Studies on EE2 have evidenced direct population-level consequences (26, 27). Moreover, endocrine disrupting chemicals (EDCs), including estrogens, that act through a similar or common mode of action can have additive effects (28–30).

Differences in sensitivity across the developmental life stages of an organism exposed to an estrogenic effluent can be a critical factor in determining the effect induced and severity of the effect. Many studies have shown that early life stages (i.e. embryonic development) can be especially vulnerable to chemical effects and these exposures can furthermore result in effects that manifest in later life stages (27, 31, 32). Due to the complexity and dynamic nature of WwTW effluent exposures, *in vitro* assays may dramatically underestimate their potential health impacts to aquatic environments. It is estimated that more than one hundred thousand xenobiotics are currently in regular use in the human population (33). Biosensor transgenic fish offer great potential for both mixture and temporal effects analyses for exposure to chemicals and their mixtures (34–36). Here we applied an Estrogen Responsive Element (ERE) – Green Fluorescent Protein (GFP) transgenic zebrafish (*Danio rerio*) model in a pigment free (Casper) strain of zebrafish [ERE-GFP-Casper; (37)] to investigate for health effects and life stage sensitivities following chronic and acute exposures to a WwTW effluent with known (measured) estrogenic content. The responses measured included assessments on survivorship, growth, sex and sexual development (via gonadal histopathology), fluorescence induction in both target tissues (via fluorescence microscopy) and in whole bodies via quantification of *gfp* mRNA (via qRT-PCR). Levels of whole body/liver *gfp* mRNA were also compared with levels of *vtg* mRNA, a well-established biomarker for estrogen exposure.

## Materials and Methods

### Test Animals and Husbandry

A transgenic zebrafish strain developed at the University of Exeter, ERE-GFP Casper [3×ERE:Gal4ff and UAS:GFP; (37)] was employed in this work. Briefly, this translucent strain was derived from crossing an established transgenic ERE-GFP line (38) with a Casper strain (containing silenced *roy* (dark) and *nacre* (silver) pigmentation genes) acquired from University College London. All zebrafish were raised under a 12-h light, 12-h dark cycle photoperiod regime with gradual dawn and dusk transitions of 30 minutes, fed to satiation three times daily with ZM Fry food 000/100 (Zebrafish Management Ltd., Winchester, U.K.) and Liquifry No. 1 (Interpet, Dorking, U.K.) up to 21 dpf, progressing to live newly hatched Brine Shrimp (Artemia) and frozen Gamma Omega 3 Enriched Brineshrimp (Zebrafish Management Ltd) from 21 dpf to adulthood.

### Water Supply

The supply of water to the laboratory dosing system was maintained using reverse-osmosis with the addition of salts [as described in (39)], to obtain system water. Water temperature and pH levels were monitored daily and ranged between 26.5 and 27.6 °C in all experiments, with pH levels between 6.8 and 7.7. Dissolved oxygen concentrations were maintained at >80% of the air saturation throughout all exposures. Conductivity ranged between 200 and 260 μS/cm.

System water and test chemical flowrates were measured twice weekly; flow-rates to each aquaria (working volume of 20 L) were set at 10 mL/min to provide just over 75% replacement every 24 hours.

### Experimental Design

**Figure 1:**
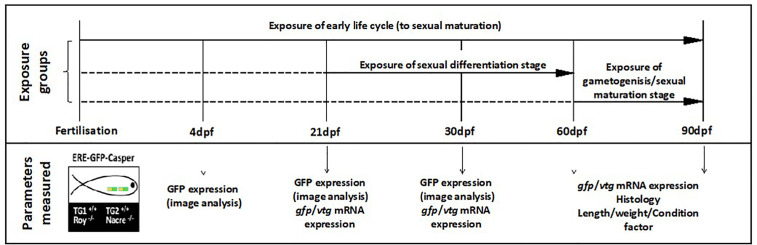
Design of the zebrafish effluent exposure experiment. Exposures were conducted to a system water control (SWC), EE2 (10 ng/L) or WwTW effluent concentrations of 100% or 50% for the different developmental life stages indicated. Exposures are indicated with solid horizontal lines with dashed lines indicating fish maintenance in clean water. There were three replicate tanks per treatment. The time points for measurement of the different endpoints are indicated. From 0-4 dpf, embryos/larvae were housed in 500 mL glass dishes without any water flow at a density of 50 fish per dish and kept in an incubator at 28 °C. From 5-21 dpf, fish were kept in 3.5 L nursery tanks placed within the 20 L glass tanks and then subsequently transferred to the 20 L tanks at 21 dpf.

ERE-TG GFP Casper zebrafish (n = 60 per treatment x 3 replicate 20L glass tanks) were exposed to either a system water control (SWC), a reference estrogen positive control (EE2 at nominal 10 ng/L) or a wastewater effluent at concentrations of either 100% or 50% for the following developmental life periods: (i) 0-90 days post fertilization (dpf) (ii) 21-60 dpf (iii) 60-90 dpf (**Figure 1**). These life stages were chosen to cover (i) the entire developmental period to sexual maturation, (ii) the period of gonadal sexual differentiation in zebrafish and (iii) gametogenesis (sexual maturation). Fish in 0-90 dpf developmental groups were exposed from within two hours of embryo collection. Fish in other developmental life stage groups were kept in SWC tanks until commencement of their exposure at either 21-60 dpf or 60-90 dpf. The 50% effluent concentration was obtained by diluting the 100% effluent 1:1 with system water. Solvent free stock solutions of EE2 (≥ 98% purity, Sigma-Aldrich, Poole, UK) were prepared every three days by adding 250 μl of a stock solution of EE2 (prepared in HPLC-grade acetone, Fisher Scientific, Loughborough, UK) to a 2.5 L clean Winchester bottle, that had been acid washed and rinsed with acetone (Analytical reagent grade, Fisher Scientific) and dichloromethane (HPLC-grade, Fisher Scientific) to remove any trace contaminants. After evaporation of the acetone, at room temperature, 2.5 L of system water was added, and the solution stirred for approximately 2 hours. The solvent free stock was then delivered to another glass mixing vessel via a peristaltic pump, to mix with the dilution water for the final exposure concentration of 10 ng/L.

### Wastewater Treatment Works Effluent

Effluent was collected twice weekly from a UK WwTW in batches of 500 L over a period of 13 weeks using a Glass Reinforced Plastic water tank. Each consignment of effluent was collected from the final effluent stream at the WwTW between the hours of 08.00 and 09.00 a.m., immediately transported back to the laboratory and then placed into a fully enclosed stainless steel holding tank chilled to 4°C, before being transferred via a peristaltic pump system into glass mixing tanks, where it was slowly acclimated to the desired test temperature of 28 °C before being pumped into the exposure tanks (**Supplementary Figure S1**). The total population equivalent (P.E., a measure of the organic strength of the effluent) or unit per capita loading for this WwTW was 141698. More details about the WwTW, including the treatment processes involved are provided in **Supplementary Table S2**. Dissolved oxygen concentrations for all consignments of effluent were above 80%, with pH values ranging between 6.5 and 7.2, and temperatures ranging from 17.3 to 20.7 °C. Full details of all physiochemical parameters measured, including dissolved/suspended solids, nitrogen/phosphorus levels, and biochemical and chemical oxygen demand (BOD/COD) concentrations, are provided in **Table 1**.

**Table 1:** Physiochemical parameters measured for the treated wastewater effluent collected from the WwTW during the different experimental periods.

Physicochemical parameter	Jun-14	Jul-14	Aug-14	Sep-14
pH	7.0 ± 0.05	6.8 ± 0.05	7.2 ± 0.05	6.8 ± 0.1
Rainfall (mm)	8.0 ± 1.4	5.5 ± 3.4	7.0 ± 0.8	2.3 ± 1.2
Temperature (°C)	20.1 ± 1.43	20.7 ± 0.87	19.3 ± 0.61	17.3 ± 0.43
Suspended solids (mg/l)	13.0 ± 0.64	14.0 ± 1.36	7.0 ± 1.15	12.0 ± 0.94
Biochemical oxygen demand (BOD; mg/l)	7.0 ± 0.76	5.0 ± 0.22	3.5 ± 0.08	3.0 ± 0.65
Chemical oxygen demand (COD; mg/l)	23.0 ± 0.32	40.0 ± 0.45	35.0 ± 0.62	68.0 ± 0.82
NH3 (mg/l)	4.6 ± 0.12	6.2 ± 0.06	<0.2 ± 0.01	4.7 ± 0.03
Cl^-^ (mg/l)	n/a	n/a	n/a	n/a
Estrone (E1; ng/L)	0.86 ± 0.86	0.07 ± 0.04	1.92 ± 0.75	19.8 ± 6.3
17β-Estradiol (E2; ng/L)	0.07 ± 0.07	0.13 ± 0.07	0.29 ± 0.08	12.8 ± 9.7
17α-Ethinylestradiol (EE2; ng/L)	<MDL	<MDL	<MDL	<MDL

To calculate monthly mean estrogen concentrations, concentrations that were over the limits of detection (≥ MDL) but below the limits of quantification (< MQL) were assigned the MDL value. Concentrations below the MDL were considered to be zero. Individual measured concentrations of E1 and E2 are presented in **Supplementary Table S3**. EE2 concentrations were below the MDL (0.5 ng/L) throughout.

Data are reported as mean ± SE.

### Fish Sampling and Analysis of Phenotypic Endpoints

For this study, triplicate tanks of fish were sampled at various time periods during development (as shown in **Figure 1**) and examined for GFP protein expression using image analysis (n = 15 fish per treatment) *vtg* and *gfp* mRNA expression (n = 15 fish per treatment) and histological analysis of gonads (n = 30 fish per treatment). Survival, fork length (to the nearest 1mm) and wet weight (to the nearest 0.01 g) were recorded for each fish. Length and weight data were used to calculate the condition factor (K-factor) for each fish using the formula: K-factor = (weight (mg)/length (mm)^-3^) × 100. Other measured endpoints included hatching rate success (at 72 and 96 hours post fertilization, hpf). Fish were sacrificed by terminal anesthesia with benzocaine (0.5 g/L; ethyl-p-aminobenzoate; Sigma-Aldrich) followed by destruction of the brain and either snap frozen in liquid nitrogen and stored at −80 °C for *vtg* and *gfp* mRNA expression analysis or fixed in toto in Bouin’s fixative (Fisher Scientific) for a maximum of 6 hours for gonadal histopathology. All animal use and protocols were carried out ethically in accordance with U.K. Home Office guidelines (Animals (Scientific Procedures) Act 1986).

### Measurement of Concentrations of Environmental Estrogens; Estrone (E1), 17β-Estradiol (E2), 17α-Ethinylestradiol (EE2)

Wastewater effluent samples (2 x 2.5 L) were collected in amber glass bottles, that had been pre-washed and acid rinsed. Acetic acid (1 %) and methanol (5 %) were added to the samples immediately after collection which were then stored at 4 °C, until extraction. Filtration was carried out within 12 h after sample collection. Three 1 L sub-samples were spiked with 100 ng deuterated estrone- (E1-d4), 100 ng deuterated 17β-estradiol (E2-d4) and 10 ng deuterated 17α-ethinyloestradiol (EE2-d4) as internal standards (ISTDs). Deuterated E1-d4 and E2-d4 were obtained from CDN Isotopes (Quebec, Canada), and EE2d4 from Cambridge Isotope Labs (Andover, MA, USA). The samples spiked with ISTD were then pre-filtered through glass wool and filter paper Whatman No1 (Whatman, Maidstone, UK) to remove any large particulates, prior to extraction onto pre-conditioned (with HPLC-grade methanol (10 mL) and HPLC-grade water (10 mL, both Fisher Chemicals) Oasis HLB cartridges (20 mL, 1 g, 60 μm particle size; Waters, Manchester, UK). Twenty mL of HPLC-grade water was used to wash the sorbent. The cartridge was then dried under vacuum and elution was performed with 10 mL of MeOH. Extracts were dried and reconstituted in 0.5 mL of methanol. The residue was then cleaned up using DSC-NH2 cartridges (Sigma-Aldrich) according to Flores et al. (40) (see *Supplementary Material* for more details). After this, extracts were reconstituted in 100 µL of water/acetonitrile (7:3, v/v) and passed through 0.22 µm centrifuge filters and stored at -80°C before analyses. Subsequent measurement of E1, E2, EE2 concentrations was performed via ultra high-performance liquid chromatography tandem mass spectrometry (UHPLC-MS/MS) using a Waters Acquity UHPLC system coupled to a Quattro Premier triple quadrupole mass spectrometer from Micromass (Waters, Manchester, UK). The method detection limits (MDL) for E1, E2 and EE2 were 0.2 ng/L, 0.4 ng/L and 0.5 ng/L, respectively. The method quantification limits (MQL) for E1, E2 and EE2 were 0.6 ng/L, 1.2 ng/L and 1.5 ng/L, respectively. Details of the LC-MS method can be found in the *Supplementary Material*. All positive controls (EE2) and controls (using HPLC-grade water) were treated and extracted under the same conditions.

### Image Analysis

Fluorescence microscopy (Zeiss Axio Observer.Z1; Zeiss, Cambridge, UK) was used to visualize GFP protein expression in the zebrafish with a 1-10x objective (for the different life stages), employing the Axiovision digital software program for the production of images. Exposure times employed were kept consistent and were dependent on the area photographed dictated by the different fluorescence intensities seen in the different target tissues, (50 ms for head region, 20 ms for mid trunk region, and 400 ms for tail section). GFP expression was measured at 4, 21 and 30 dpf, respectively (n = 15 per treatment x 3 replicates). Prior to live imaging, all fish were anesthetized with 0.4% tricaine, mounted in 4% methylcellulose in a glass-bottom dish and oriented in dorsal, ventral and lateral views. All images were aligned, and the contrast adjusted using Adobe Photoshop CS4 (Adobe Systems, San Jose, CA, USA) keeping the same intensity for all samples.

### Histological Analysis

At each time point analyzed, a minimum of 30 zebrafish per treatment were fixed and sectioned to determine both gonadal sex and gonadal maturity and to assess for any alterations in the structural organization of the gonad (**Supplementary Figure S2**), e.g., intersex. Details on the histological processing methods are provided in the *Supplementary Material*. The stage of gonadal maturity was recorded according to the standardized criteria described in the OECD Histopathology Guidance document (41) and using a numerical staging system (**Supplementary Table S4**).

### Quantification of the Relative Transcript Levels of *gfp* and *vtg* Using Real-Time Quantitative Polymerase Chain Reaction

#### RNA Extraction and cDNA Synthesis

The zebrafish genome, contains at least 7 *vtg* genes (42) and there is evidence that their expression varies over different life stages (42–44) which may have a bearing on life stage sensitivities to *vtg* mRNA induction. We chose *vtg1* mRNA as previous work has suggested that it is the most ubiquitously expressed *vtg* gene transcript (45). Whole body samples (for 21, 30 and 60 dpf fish) and liver samples (90 dpf) for *vtg* and *gfp* mRNA expression analysis were immediately snap-frozen in liquid nitrogen upon collection and stored at −80 °C until use. Total RNA was extracted from all samples using Tri Reagent (Sigma), according to the manufacturer’s instructions. The amount of RNA was quantified using a NanoDrop 1000 spectrophotometer and RNA purity determined through the measurement of the A260/A280 and A260/A230 ratios, respectively. Reverse transcription was subsequently carried out by incubating 1 µg RQ1 DNase treated (Promega, Southampton, UK) total RNA with 5 mM random hexamers (Eurofins MWG Operon. Ebersberg, Germany), 10 mM dNTPs and 1 µl MMLV-Reverse transcriptase (Promega) in the appropriate buffer, following the manufacturer’s instructions.

#### RT-qPCR

RT-qPCR was carried out on cDNA samples for each treatment group and sampling point for selected target genes with the iCycler iQ Real-time Detection System (Bio-Rad Laboratories Inc., CA, USA). Gene-specific primer sequences were designed with Beacon Designer 7.2 software (Premier Biosoft International, Palo Alto, USA) and obtained from Eurofins MWG Operon. In brief, primer pairs were optimized for annealing temperature (Ta), specificity confirmed by melt curve analysis, and the detection range, linearity and amplification efficiency (E) established using serial dilutions of zebrafish cDNA, as described previously (46). The primer sequences, PCR product sizes, annealing temperatures and PCR efficiencies for each primer pair are shown in the *Supplementary Material* (**Supplementary Table S5**). RT-qPCR was carried out using iTaq Universal SYBR Green Supermix (Bio-Rad), with an initial activation step of 95 °C for 15 min followed by 40 cycles of denaturation (95 °C, 10 s) and annealing (45 s at the appropriate Ta) and final melt curve analysis. Expression levels of ribosomal protein L8 (*rpl8*) were used as a ‘housekeeping’ gene to normalize the expression of other genes and determine efficiency-corrected relative expression levels, as described previously (46). All samples were analyzed in triplicates and a template-minus negative control was run on each plate to verify the absence of DNA contamination.

### Statistical Analysis

In all cases, data were checked for normality using the D’Agostino-Pearson test and for homogeneity of variance using Bartlett’s or Levene’s test prior to statistical analysis. Data meeting these tests were analyzed using parametric tests, e.g., analysis of variance (ANOVA) procedures, whilst data that did not conform to normality were subject to nonparametric tests, e.g., Kruskal–Wallis. Post hoc analysis was performed against controls using the Dunnett’s test and with the Tukey test for between-treatment comparisons. Data were transformed, where necessary, using log10. Any significant differences from expected sex ratios or stages of gonadal development were tested using the chi-square test. The relationship between levels of *gfp* vs *vtg* expression were measured using a 2 tailed Pearson’s correlation test. All statistical analyses were run in GraphPad Prism 7 (GraphPad Software, Inc., San Diego, USA). Statistical significance was accepted as p <0.05 for all comparisons and values are quoted as mean values and ranges as standard error (SE). To calculate monthly mean estrogen concentrations, concentrations that were over the limits of detection (≥ MDL) but below the limits of quantification (< MQL) were assigned the MDL value. Concentrations below the MDL were considered to be zero (47).

## Results

### Concentrations of Steroid Estrogens in the Exposure Effluent

Analytical measurements for E1, E2 and EE2 were used to characterize the estrogenic content of the effluent (**Table 1** and **Supplementary Table S3**). Measured concentrations of E1 from the different consignments of effluent collected from the WwTW ranged from below MDL (0.2 ng/L) to 4.8 ng/L during June 2014 (mean 0.86 ± 0.86 ng/L) up to between 2.7 and 39.6 ng/L during Sept 2014, (mean 19.8 ± 6.26 ng/L). The mean concentration of E1 measured over the experimental period was 5.3 ± 2.0 ng/L). E2 concentrations in the different consignments were below MDL (0.4 ng/L) between June and August and were overall higher in September, varying between <MQL (1.2 ng/L) and 46.4 ng/L. The mean concentration of E2 measured over the experimental period was 2.5 ± 1.8 ng/L). EE2 concentrations were <MDL (0.5 ng/L) in all effluent consignments.

Measured concentrations of EE2 in the positive control tanks were between 70% and 120% of nominal concentrations (mean 8.6 ± 3.6 ng/L; data not shown). Concentrations of E1, E2 and EE2 in all control tanks were below the MDL i.e., 0.2; 0.4; 0.5 ng/L, respectively, and were ascribed as zero.

### Phenotypic Endpoints

#### Survival and Hatching Success

**Figure 2:**
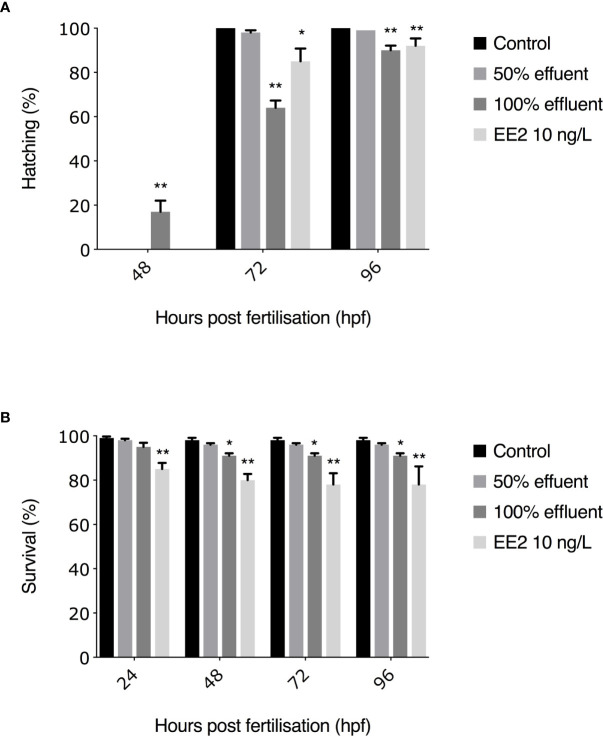
Hatching rates **(A)** and subsequent survival **(B)** to 96 hpf of ERE-GFP Casper zebrafish. Transgenic zebrafish embryos were exposed to EE2 (10 ng/L), a WwTW effluent concentration (50% or 100%) or dilution water control. Data are reported as mean ± SE. Asterisks denote a significant difference (*p ≤ 0.05, **p ≤ 0.01) compared with control.

For the 0-90 dpf exposure, lower survival rates were observed in EE2-exposed embryos at 24 hpf (460/540 = 85%, p ≤ 0.01, **Figure 2B**). At 48 hpf, survival rates were significantly lower compared with untreated controls in embryos exposed to 100% effluent and EE2 (493/540 = 91%, p ≤ 0.05, 434/540 = 80%, p ≤ 0.01, respectively, **Figure 2B**). At both 72 and 96 hpf, survival rates of embryos exposed to 100% effluent and EE2 were significantly lower compared to controls (493/540 = 91%, p ≤ 0.05, 419/540 = 78%, p ≤ 0.01, **Figure 2B**).

All surviving embryos in the 0-90 dpf experiment in control groups hatched by 72 hpf. The earliest observed hatching occurred at 48 hpf in the 100% WwTW effluent exposure (82/493 = 17%, p ≤ 0.01). At 72 hpf, hatching rates were significantly lower in embryos exposed to 100% effluent (317/493 = 64%, p ≤ 0.01) and EE2 (358/419 = 85%, p ≤ 0.05 compared to controls. At 96 hpf, hatching rates differed significantly in the 100% effluent and EE2 treatments compared with untreated controls (445/493 = 90%, p ≤ 0.01, 385/419 = 92%, p ≤ 0.01, respectively, **Figure 2A**). Post-hatch survival in all treatment groups was >80%, thus exceeding OECD guidelines of ≥70% survival for controls (48), and with no statistically significant differences between treatments (data not shown).

Survival in treatment groups for exposures between 21-60 days and 60-90 days was >80% throughout and there were no statistically significant differences between treatments (data not shown).

#### Body Size/Condition Factor

Male and female body weight, length and condition factor were measured at 60 dpf and 90 dpf for all exposures (see **Table 2**).

**Table 2:** Length, weight, and condition factor (*K*-factor) determined in male and female zebrafish in control (dilution water), EE2 (10 ng/l) or WwTW effluent (50% or 100%) exposures across different developmental life stages.

Window of exposure	Treatment	n	Length (mm)		Weight (g)		*K*-factor	
			Females	Males	Females	Males	Females	Males
0 – 90 dpf *Age: 60 dpf*	Control	30	27 ± 0.5	23 ± 0.6	0.3 ± 0.01	0.2 ± 0.01	1.8 ± 0.08	1.8 ± 0.1
	50% effluent	30	27 ± 0.5	25 ± 0.6	0.4 ± 0.03*	0.3 ± 0.01	1.8 ± 0.1	1.6 ± 0.06
	100% effluent	30	29 ± 0.4**	27 ± 0.7**	0.5 ± 0.02**	0.4 ± 0.02**	1.8 ± 0.06	1.6 ± 0.09
	EE2 (10 ng/L)	30	24 ± 0.5**	19 ± 1.1**	0.2 ± 0.01**	0.1 ± 0.03**	1.6 ± 0.03	1.8 ± 0.08
0 – 90 dpf *Age: 90 dpf*	Control	36	36 ± 1.6	35 ± 0.5	0.6 ± 0.02	0.4 ± 0.01	1.5 ± 0.3	1.0 ± 0.04
	50% effluent	36	38 ± 1.2*	37 ± 0.6*	0.7 ± 0.04*	0.5 ± 0.02*	1.1 ± 0.06	0.9 ± 0.02
	100% effluent	36	40 ± 1.1*	38 ± 0.7**	0.7 ± 0.02*	0.5 ± 0.03*	1.2 ± 0.08	0.9 ± 0.03
	EE2 (10 ng/L)	36	33 ± 1.0**	30 ± 0.7**	0.5 ± 0.04**	0.3 ± 0.02	1.5 ± 0.1	1.0 ± 0.03
21 – 60 dpf *Age: 60 dpf*	Control	36	27 ± 0.5	24 ± 0.6	0.4 ± 0.01	0.3 ± 0.01	1.8 ± 0.08	1.8 ± 0.1
	50% effluent	36	30 ± 0.2**	25 ± 0.5	0.4 ± 0.02	0.3 ± 0.02	1.7 ± 0.09	1.7 ± 0.05
	100% effluent	36	33 ± 0.3**	26 ± 0.7*	0.5 ± 0.01*	0.2 ± 0.1	1.0 ± 0.03**	1.4 ± 0.09**
	EE2 (10 ng/L)	36	26 ± 0.4*	21 ± 0.4**	0.3 ± 0.01*	0.2 ± 0.01*	1.2 ± 0.04**	1.2 ± 0.07**
60 – 90 dpf *Age 90 dpf*	Control	36	36 ± 0.5	35 ± 0.3	0.6 ± 0.01	0.4 ± 0.01	1.5 ± 0.0	1.0 ± 0.03
	50% effluent	36	33 ± 0.6*	30 ± 0.5**	0.5 ± 0.03	0.3 ± 0.01	2.0 ± 0.1	1.5 ± 0.08
	100% effluent	36	31 ± 0.5**	29 ± 0.5**	0.4 ± 0.03*	0.3 ± 0.02**	1.8 ± 0.1*	1.4 ± 0.08*
	EE2 (10 ng/L)	36	28 ± 0.8**	29 ± 0.8**	0.4 ± 0.04**	0.4 ± 0.02	1.9 ± 0.09	1.9 ± 0.07**

Data are reported as mean ± SE. Asterisks denote a significant difference (*p < 0.05, **p < 0.01) compared with system water control.

##### Females

At 60 dpf, the length and weight of females in the 0-90 dpf exposure were higher in 100% effluent (p ≤ 0.01) and lower in females exposed to EE2 (p ≤ 0.01) compared with controls. Females exposed to 50% effluent were heavier than untreated controls (p ≤ 0.05). At 90 dpf, length and weights in female fish were higher for exposure to both 50% and 100% effluent (p ≤ 0.05), but lower in females exposed to EE2 (p ≤ 0.01) compared with controls. No differences were seen in K-factor in any of these treatment groups.

For the 21-60 dpf exposure, females at 60 dpf were greater in length (p ≤ 0.01) and heavier (p ≤ 0.05) in 100% effluent treatments, greater in length in the 50% effluent (p ≤ 0.01) and shorter in length and smaller in weight in the EE2 exposure (both p ≤ 0.05) compared with controls. K-factor in females was lower in both 100% effluent and EE2 treated fish (p ≤ 0.01).

For exposures carried out between 60-90 dpf, females at 90 dpf were shorter in length and weighed less in the 100% effluent (p ≤ 0.01, length and p ≤ 0.05, weight) and EE2 treatments (both p ≤ 0.01), and shorter in length in 50% effluent exposure (p ≤ 0.05). Condition factor was higher in females exposed to 100% effluent compared to controls (p ≤ 0.05).

##### Males

In the 0-90 dpf continuous exposure, at 60dpf both length and weight of males were significantly higher in the 100% effluent exposure groups (p ≤ 0.01) and lower in males exposed to EE2 (p ≤ 0.01) compared to untreated controls and these effects on length persisted at 90 dpf. There was no observed effect on growth in males exposed to 50% effluent at 60 dpf, but at 90 dpf they were larger than in controls (p ≤ 0.05). No significant differences in K-factor were found in any of the treatment groups at either time point for this exposure regime.

For the exposure during the period of sexual differentiation (21-60 dpf), males exposed to 100% effluent were greater in length (p ≤ 0.05) compared to controls whereas males exposed to EE2 were shorter in length (p ≤ 0.01) and smaller in weight (p ≤ 0.05). No effects on growth were observed in the 50% effluent exposure group. Males in both 100% effluent and EE2 exposures (p ≤ 0.01) also had a lower K-factor compared with controls.

For exposures carried out from 60-90 dpf, males at 90 dpf in 100% effluent exposures were shorter in length and smaller in weight (p ≤ 0.01) and smaller in length in the 50% effluent and EE2 exposures (p ≤ 0.01). Males exposed to 100% effluent (p ≤ 0.05) and EE2 (p ≤ 0.01) had a higher K-factor compared to controls.

#### Sex Ratio

**Figure 3:**
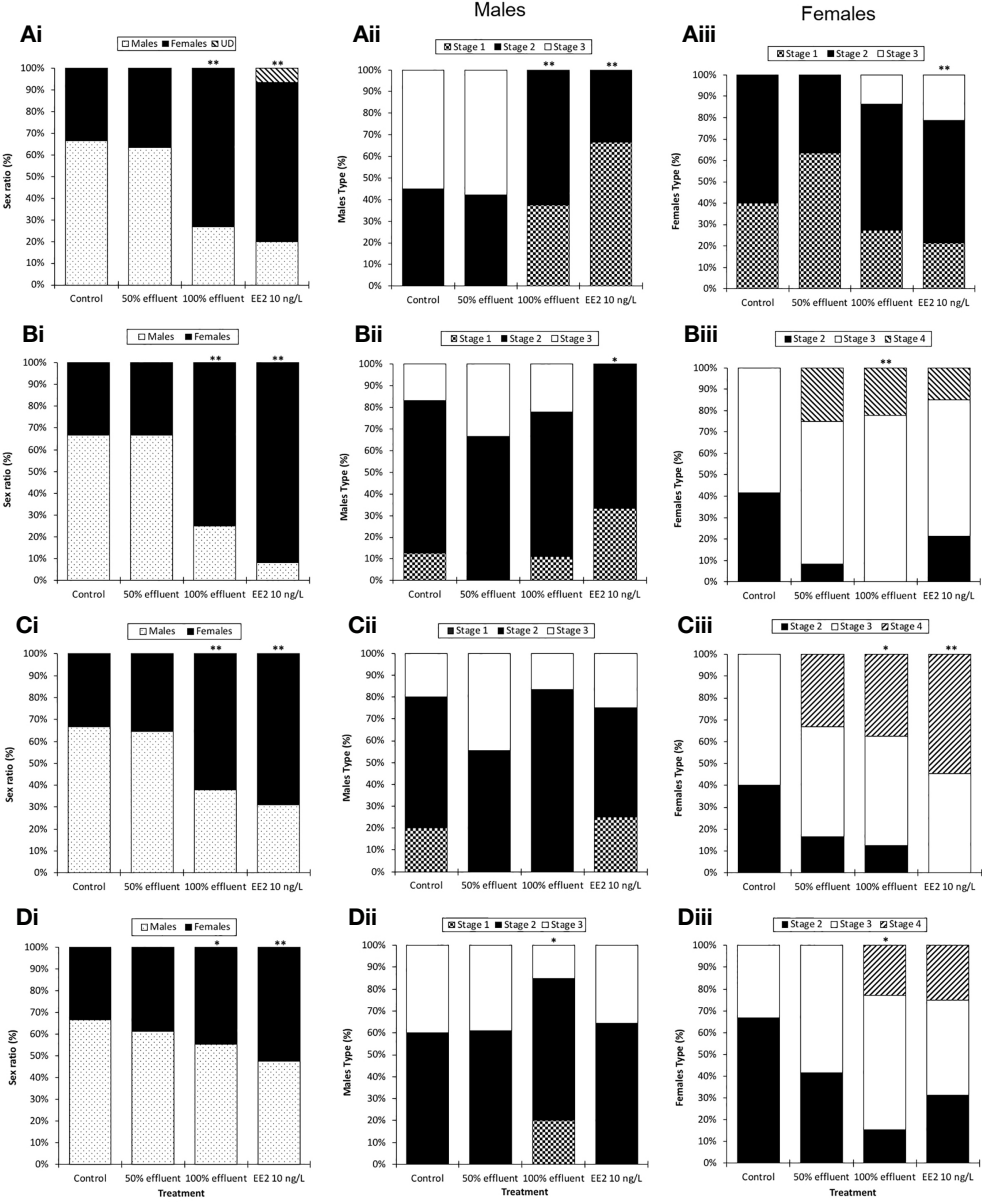
Sex ratios and male and female maturity index in ERE-GFP Casper zebrafish. Sex and indices of sexual maturity were measured in transgenic zebrafish at 60 dpf or 90 dpf following exposure to EE2 (10 ng/L), a WwTW effluent (at 100% or 50% dilution) or a dilution water control during the following developmental periods: **(A)** 0-90 days (data analysis at 60 dpf); **(B)** 0-90 days (data analysis at 90 dpf) **(C)** 21-60 days (data analysis at 60 dpf) and **(D)** 60-90 days (data analysis at 90 dpf). Data are reported as mean ± SE. Asterisks denote statistically significant differences (*p<0.05, **p<0.01) from expected sex/maturity index ratios according to a chi-square contingency table.

In the 0-90 dpf exposure group, at 60 dpf there was a sex ratio bias in favor of males in both controls and the 50% effluent (around 2:1 male to female (**Figure 3Ai**). In contrast, in the exposures to 100% effluent and EE2, at 60 dpf the sex ratios were 1:3 male to female (χ²(1, n = 120) =9.64, p ≤ 0.01) and 1:4 male to female (χ²(1, n = 120) =14.04, p ≤ 0.01), respectively. A proportion of fish in EE2 exposure groups (7%) had not undergone sexual differentiation (and/or the sex could not be identified). The sex ratio at 90 dpf were similar to those at 60 dpf with 2:1 male to females in controls and the 50% effluent exposure and 1:3 male to female ratio in fish exposed to 100% effluent (χ²(1, n = 144) =12.59, p ≤ 0.01). At 90 dpf in the EE2 exposure, there was an enhanced feminized effect compared with at 60 dpf (1:11 male to females, (χ²(1, n = 144) =26.13, p ≤ 0.01, **Figure 3Bi**).

For the groups exposed during the period of sexual differentiation (21-60 dpf), sex ratios at 60 dpf in both controls and the 50% effluent were the same as for the 0- 90 dpf exposures, i.e. a 2:1 male to female ratio, for fish exposed to 100% effluent, 1:1.75 male to female (χ²(1, n = 120) = 7.52, p ≤ 0.01) and for EE2 1:2.1 male to female (χ²(1, n = 120) = 11.38, p ≤ 0.01) (**Figure 3Ci**).

For fish exposed during 60-90 dpf, at 90 dpf the sex ratio in the control group was 2:1 male to female, 3:2 male to female in the 50% effluent exposure, 5:4 male to female in the 100% effluent (χ²(1, n = 165) = 12.59, p ≤ 0.05) and 6:7 male to female in the EE2 (χ²(1, n = 165) = 26.13, p ≤ 0.01) exposure (**Figure 3Di**).

#### Sexual Differentiation and Development

##### Males

Continuous exposure (0-90 dpf) to either 100% effluent (χ²(2, n = 28) = 12.2, p ≤ 0.01) or EE2 (χ²(2, n = 26) = 16.8, p ≤ 0.01) appeared to delay testis maturation; at 60 dpf none of the testes had progressed beyond stage 2, whereas 55% of control males were at stage 3 (**Figure 3Aii**). At 90 dpf, however, testis development differed only in the EE2 exposure treatment where it was still delayed (none had reached stage 3, (χ²(2, n = 26) = 6.9, p ≤ 0.05) (**Figure 3Bii**).

For the exposure during 21-60 dpf there was no effect on testis development at 60 dpf in any of the treatment groups (**Figure 3Cii**). Exposure during 60-90 dpf resulted in a delayed testis development in males exposed to 100% effluent (χ²(2, n = 37) = 6.3, p ≤ 0.05), where only 15% of males were at stage 3 versus 40% in the respective controls (**Figure 3Dii**). Intersex gonads (i.e., testis-ova) were not observed in any treatment group.

##### Females

In contrast with males, in females sampled at 60 dpf in the 0-90 dpf exposure, EE2 appeared to have an enhanced effect on ovary maturation (χ²(2, n = 38) = 3.1, p ≤ 0.01; 21% were at stage 3, whereas in controls none were more advanced than stage 2 (**Figure 3Aiii**). There was a tendency also for this to be the case in females exposed to 100% effluent (14% at stage 3), but this was not significant. At 90 dpf, females exposed to 100% effluent (χ²(2, n = 39) = 14.3, p ≤ 0.01) had a higher ovary maturity index compared with controls (22% classed as at stage 4 versus none in controls (**Figure 3Biii**). Stage 4 ovaries were also observed in EE2 (15%) and 50% effluent (33%) exposures, but they did not differ statistically from controls.

In females exposed during 21-60 dpf, there was an effect on ovary development in both the 100% effluent (χ²(2, n = 28) = 8.1, p ≤ 0.05) and EE2 (χ²(2, n = 26) = 9.3, p ≤ 0.01) exposures (**Figure 3Ciii**). In females exposed to 100% effluent during 60-90 dpf, at 90 dpf, ovary development was more advanced compared with the respective controls (χ²(2, n = 23) = 7.9, p ≤ 0.05); 23% of females were classed as at stage 4 whereas none were this advanced in the respective controls (**Figure 3Diii**). Some females in the 60-90 dpf exposure to EE2 were also at stage 4 of ovarian development but this was not statistically different from the controls.

### GFP Tissue Expression

**Figure 4:**
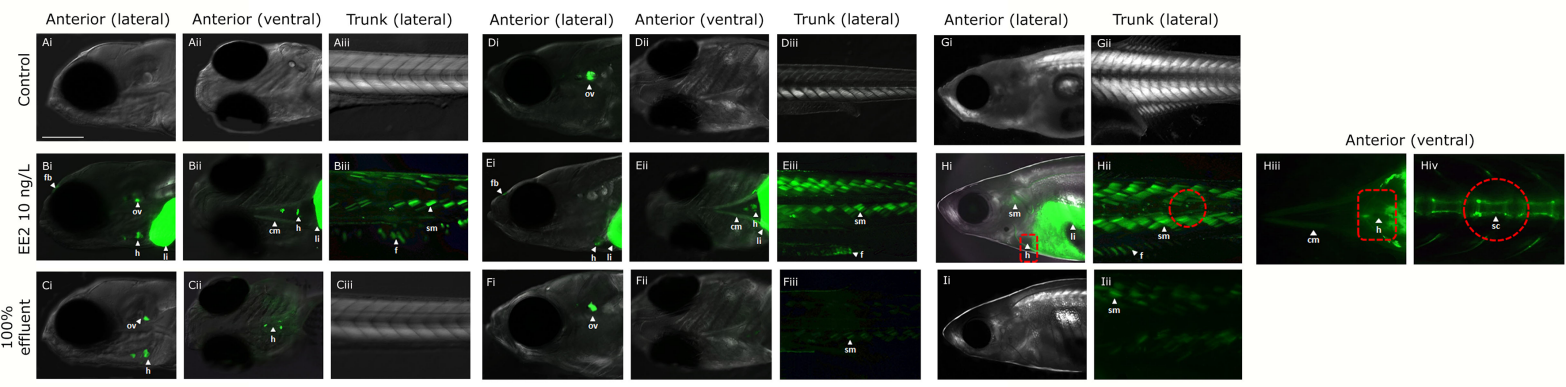
Expression of GFP in body tissues in ERE-GFP Casper zebrafish exposed to EE2 and undiluted WwTW effluent from 0-90 dpf. Transgenic zebrafish at 4 dpf in dilution water control **(A)**, EE2 (10 ng/L, positive control, **B**), and wastewater effluent (100%) exposed groups **(C)**; at 21 dpf in system water control, **D**), EE2 (10 ng/L, **E**) and 100% wastewater effluent **(F)**; at 30 dpf in system water control **(G)**, EE2 (10 ng/L, **H**) and 100% wastewater effluent **(I)**. GFP expression is shown for regions in the head (lateral – i and ventral – ii views) and trunk (lateral view - iii). GFP induction was observed in the cranial muscles (cm), fin (f), forebrain (fb), heart (h), liver (li), otic vesicle (ov), skeletal bone (sc) and somite muscles (sm) in the EE2 exposed fish **(B, E, H)**, and heart, liver and otic vesicle in wastewater effluent exposed fish **(C, F, I)**. Bar = 100 μm.

In control fish, a weak basal GFP expression was only observed in the otic vesicle at 21 dpf (**Figure 4Di**) but there was no GPF protein expression in any other tissue at any other time point (**Figures 4A, D, G** **)**.

#### Continuous Exposure From Fertilization

In fish continuously exposed to full strength effluent between 0-90 dpf, GFP expression varied across the different developmental time-points. At 4 dpf, GFP expression was seen in the heart and otic vesicle (**Figures 4Ci, ii**), in 21 dpf fish, expression of GFP occurred in the otic vesicle and somites (**Figures 4Fi–iii**) and, at 30 dpf, GFP expression was observed in the somites only (**Figure 4Iii**). In fish continuously exposed to EE2, GFP expression was observed in the cranial muscle, fin, forebrain, heart, liver, otic vesicle and somites (**Figures 4Bi–iii**) at 4 dpf with comparable findings at 21 dpf (except for expression in the otic vesicle, **Figures 4Ei–iii**). At 30 dpf, GFP expression in the EE2 treatment was seen in the heart, liver, skeletal tissue, fin and somites (**Figures 4Hi–iv**).

#### Exposure During Period of Gonadal Sexual Differentiation

In fish exposed to full strength effluent between 21-60 dpf, GFP expression was observed in the otic vesicle only at 30dpf with no specific GFP expression detected in 50% effluent exposures (data not shown). Tissue responses for EE2 at 30 dpf (9 days of exposure) were comparable to those for the 0-90 dpf exposure groups (data not shown).

In fish older than 30 dpf, accurate quantification of GFP expression via fluorescent microscopy was not possible due to skin thickness and quenching of the fluorescence signal (data not shown).

### mRNA Expression of *gfp* and *vtg*

**Figure 5:**
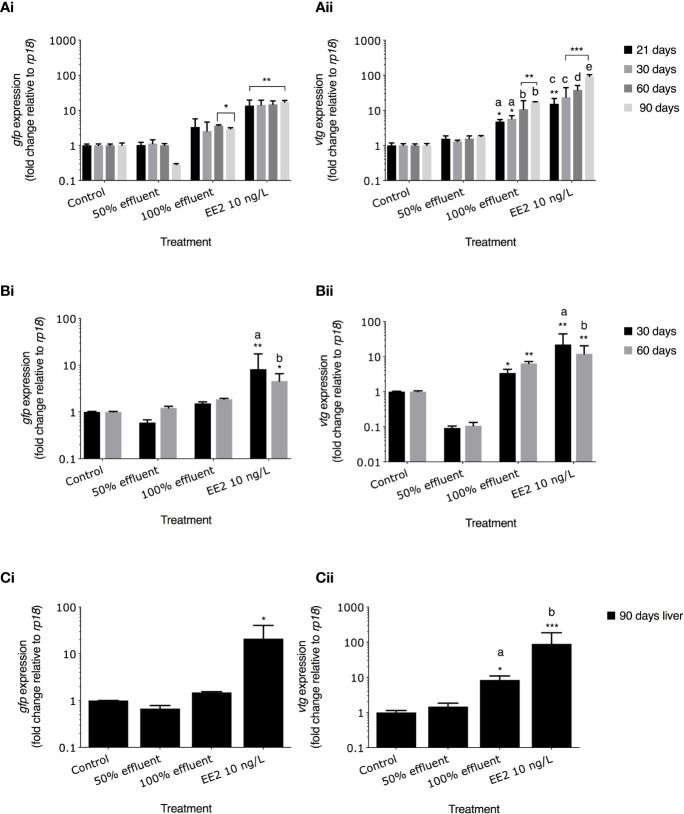
*gfp* mRNA and *vtg* mRNA levels in ERE-TG Casper zebrafish. Levels of *gfp* mRNA and *vtg* mRNA were measured in transgenic zebrafish exposed to EE2 (10 ng/L) a WwTW effluent (50% or 100%) or system water control during the developmental periods of **(A)** 0-90 days, **(B)** 21-60 days, and **(C)** 60-90 days. Data are reported as mean ± SE and expressed as fold-change compared with the control. Relative mRNA expression was determined as the ratio or target gene mRNA/*rpl8* mRNA. Asterisks denote a significant difference (*p ≤ 0.05, **p ≤ 0.01, *** p ≤ 0.001) compared with control. Within groups, different letters denote a significant difference (p ≤ 0.05).

*gfp* and *vtg* mRNA induction were measured in whole bodies at 21 dpf and 30 dpf and in the liver in 60 dpf and 90 dpf zebrafish. In fish continuously exposed to EE2 from 0-90 dpf, *gfp* mRNA levels were higher at all time points compared with controls (21 dpf,13.6-fold induction, 30 dpf, 14.3-fold induction, 60 dpf, 14.8-fold induction, 90 dpf,17-fold induction respectively, p ≤ 0.01 **Figure 5Ai**). Levels of *gfp* mRNA in 100% effluent exposures were higher at 60 dpf and 90 dpf compared with their respective controls (3.6-fold and 2.9-fold respectively, p ≤ 0.05), but there was no *gfp* mRNA induction at any time point in 50% effluent exposure groups (**Figure 5Ai**). Levels of *vtg* mRNA in fish exposed to EE2 in the 0-90 dpf exposure were 15.4-fold higher at 21 dpf (p ≤ 0.01), 23.9-fold higher at 30 dpf (p ≤ 0.001), 38.2-fold higher at 60 dpf (p ≤ 0.001), and 93.2-fold higher at 90 dpf (p ≤ 0.001) than in their respective controls (**Figure 5Aii**). Levels of *vtg* mRNA in 100% effluent exposures were higher at 60 dpf and 90 dpf compared with their respective controls (10.8-fold and 17.3-fold respectively, p ≤ 0.05 **Figure 5Aii**). There were no effects on levels of *vtg* mRNA in the 50% effluent exposure.

For exposures carried out during 21-60 dpf in the EE2 treatment *gfp* mRNA levels were elevated above controls at 30 dpf and 60 dpf (8.2-fold induction, p ≤ 0.01, and 4.5-fold induction p ≤ 0.05, respectively **Figure 5Bi**). *vtg* mRNA levels in EE2 treatments were 22.1-fold higher and 11.9-fold higher at 30 dpf and 60 dpf, respectively compared with controls (p ≤ 0.01 for both; **Figure 5Bii**). Exposure to full strength effluent did not induce *gfp* mRNA expression (**Figure 5Bi**). In the 100% effluent treatment, levels of *vtg* mRNA were 3.4-fold (30 dpf) and 6.4-fold (60 dpf) higher compared with their respective controls (p ≤ 0.05 and p ≤ 0.01, respectively; **Figure 5Bii**). mRNA levels in 50% effluent exposure groups were lower in 30 dpf (*gfp*) and both 30 and 60 dpf (*vtg*) fish (**Figure 5Bi/ii**). Levels of both *gfp* and *vtg* mRNA in 30 dpf fish exposed to EE2 differed compared to 60 dpf fish in the same treatment (p ≤ 0.05 and p ≤ 0.01, **Figure 5Bi/ii**).

For exposures carried out to EE2 during 60-90 dpf, levels of *gfp* mRNA at 90 dpf were higher in the liver when compared with untreated controls (21-fold induction, p ≤ 0.05, **Figure 5Ci**) but there were no differences for any of the other treatments. *vtg* mRNA in the liver was higher in the EE2 exposure (89.3-fold, p ≤ 0.001, Fig. 5Cii) and 100% effluent exposure (8.4-fold induction, p ≤ 0.05) compared with controls.

**Figure 6:**
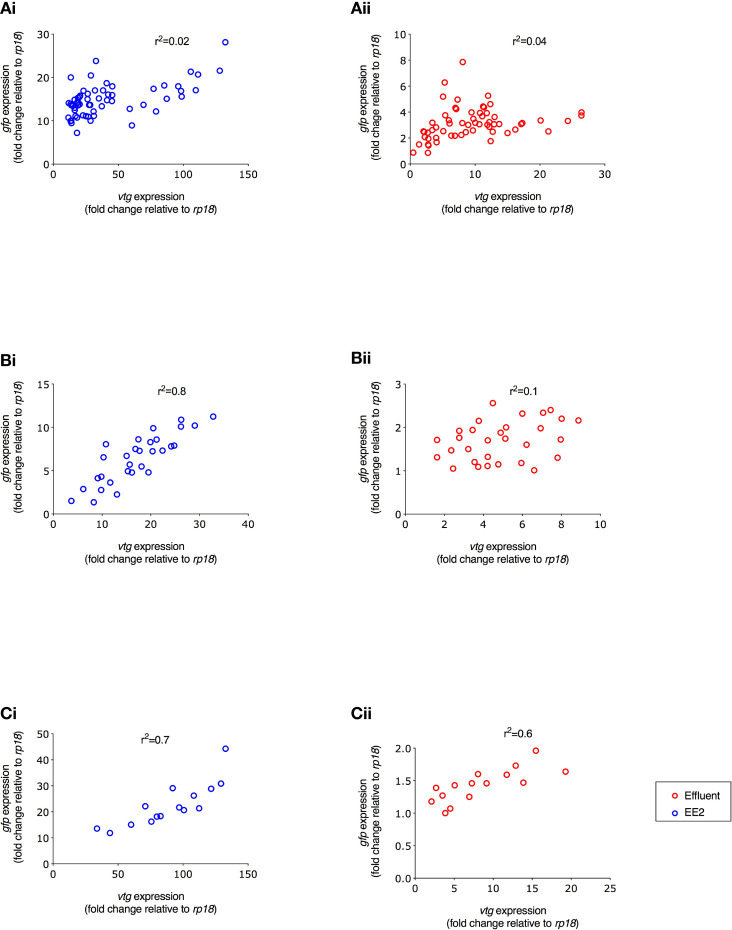
Correlation between *gfp* and *vtg* mRNA expression in ERE-GFP Casper zebrafish for different life stage exposure periods. Transgenic zebrafish were exposed to (i) EE2 (10 ng/L) or (ii) a graded effluent (50% and 100%) during the following developmental periods: **(A)** 0-90 days, **(B)** 21-60 days, and **(C)** 60-90 day. Data are reported as mean ± SE and expressed as fold-change compared with the control. Relative mRNA expression was determined as the ratio of target gene mRNA/*rpl8* mRNA.

A comparison of the induction levels of *gfp* mRNA against *vtg* mRNA in the ERE-GFP transgenic zebrafish showed that the magnitude for responses in *vtg* induction was higher compared to *gfp* for all treatments. Comparison of the data as a whole revealed a positive correlation between the levels of *gfp* expression and the levels of *vtg* expression with differing degrees of strength of these associations for both the WwTW effluent and EE2 and the developmental stages studied. For the continuous exposure over 0-90 days, there was a weak positive correlation observed for EE2 and no correlation for the effluent treatments (r^2^ = 0.02 and 0.04, respectively; **Figure 6Ai/ii**). In fish exposed to EE2 during 21-60 dpf, there was a strong positive correlation (r^2^ = 0.8, p ≤ 0.001), but a weak relationship was seen for the effluent treatments (r^2^ = 0.1, **Figure 6Bi/ii**). For exposure during 60-90 dpf, at 90 dpf (liver) there was a strong positive correlation again in the EE2 treatment (r^2^ = 0.7, p ≤ 0.001, **Figure 6Ci**), and a moderate positive correlation in effluent treatments (r^2^ = 0.6, **Figure 6Ci**i).

Full details of the measured levels of *gfp* mRNA against *vtg* mRNA induction, in whole embryo homogenates (21 and 30 dpf) and in the liver of adult zebrafish (60 and 90 dpf), for the different time points measured during all exposure periods can be found in the *Supplementary Material* (**Supplementary Figure S3**).

## Discussion

Applying the transgenic ERE-GFP Casper zebrafish to study effects of chronic exposure to an estrogenic WwTW effluent, we found both sex and developmental stage specific exposure effects on somatic growth and sexual differentiation, including a skewed sex ratio in favor of females. Target tissues for estrogenic responses (indicated by GFP protein induction) were dependent on the timing of the exposure and levels of *gfp* and *vtg* mRNA induction reflected the measured concentrations of steroidal estrogens in the effluent.

### Estrogenic Content of the Effluent

The mean WwTW effluent concentrations of the steroidal estrogens, E1, E2 and EE2, in the 0-90 dpf exposure (5.33 ± 2.01 ng/L, 2.51 ± 1.79 ng/L and <MDL, respectively) were comparable with that reported for other weakly estrogenic effluents discharging into UK rivers (49, 50) and they varied widely over the 4 months of study. The lower concentrations of steroidal estrogens, occurring in June to August may be due an enhanced rate of steroid biodegradation associated with higher temperatures and/or a higher rate of dilution that occurred due to the heavier rainfall occurring at these times. Other factors, such as the amount of organic matter (quantified by BOD and total suspended solids [TSS]), can affect the bioavailability of steroids for uptake into fish, and these were seen to differ, with a lower BOD recorded in the wastewater effluent during September compared to the summer months (June to August). The temporal variability in the steroidal estrogen content of the WwTW effluent is concordant with other reports in the literature (51, 52) and highlights the need for multiple measures for assessing chronic exposure effects and the potential for estrogenic effects in wildlife living in effluent receiving rivers.

### Biological Responses in the ERE-GFP Zebrafish Model

#### Survival and Hatching Success

We found effects on survival for exposure to EE2 only during early development to 96 hpf - a period that includes late gastrulation and early organogenesis - indicating a higher sensitivity to estrogen exposure during this life period. Similarly, the only effects of the WwTW effluent exposure on survival rate (reduced) occurred for early life stages (48 to 96 hpf), supporting previous findings (50, 53–57) and at a time when the chorion is most permeable to chemical uptake (58). The reduced rates of hatching in the exposure to EE2 and the full strength (100%) effluent also concur with other studies (59–61) and may relate to the inhibitory effects of estrogens and estrogen receptor modulators on proteolytic hatching enzymes [i.e. choriolysin; (62–64)].

#### Effects on Somatic Growth

Chronic exposure to EE2 (0-90 dpf) resulted in a reduced somatic growth in both males and females, as reported by others (65–68). We further show, however, that even relatively short-term exposures during the period of sexual differentiation (21-60 dpf) and gametogenesis/sexual maturation (60-90 dpf) reduced somatic growth in both sexes. In the literature, lower level EE2 exposures (0.1 to 4 ng/L) have been shown to have growth-promoting capacity in fish (55, 56, 69, 70). Both the longer (0-90 dpf) and shorter -term (21-60 dpf) exposures to the WwTW effluent at full strength enhanced growth in both male and female zebrafish (seen also in the roach, *Rutilus rutilus*; 9, 71). This effect may be due to additional nutrition derived from foodstuffs present in the effluent and/or potentially the presence of growth promoting chemicals. Certain pharmaceuticals found in WwTW effluents (such as oxytetracycline) have been reported to enhance body weight gain in fish (72). In contrast, exposure to 100% effluent during 60-90 dpf resulted in a decrease in somatic growth. This may relate to the higher estrogenic activity in the effluent at this time, but equally may be attributable to general toxicity effects caused by other chemicals present in the effluent (e.g., copper and cobalt, commonly found in WwTW effluents have been shown to reduce growth rates in fish (73, 74).

We found females were larger than males in all exposure groups consistent with sexual dimorphic growth patterns and body shape that occurs in zebrafish (75, 76). The growth effects of EE2 exposure seen on males may have indirectly affected rates of sexual development (in this instance a delay in gonadal maturation) as rates of sexual development can show both dependency on growth (65, 77, 78) and size in fish. Importantly, body size in fish has a positive relationship with survival and fecundity (79) and thus, a reduced body mass could reduce lifetime fitness (i.e. the total number of offspring produced during an individual’s lifetime), with potential implications for populations (80).

#### Effects on Sex and Gonadal Development

We found a clear bias towards females in all 100% effluent and EE2 exposure scenarios (compared with system water controls), which is consistent with other studies for exposure to EE2 and other steroidal estrogens present in WwTW effluents (68, 81–84). In some other fish species, full sex reversal has been reported for chronic exposure to full strength WwTW effluent. For example, in roach chronic exposure to a full-strength wastewater effluent led to sex reversal in almost all males within the population (8), and demasculization occurred in adult fathead minnows (*Pimephales promelas*) exposed to a WwTW effluent, with an estrogenic potency content averaging 50 ng 17β−estradiol equivalents (85).

In most teleost fish, the gonads maintain bipotentiality, even after gonadal differentiation (86), and thus there is a potential for exposure to exogenous estrogens at later life stages to cause sex reversal, and even in adults. Several studies, however, have shown that sex reversal in zebrafish is highly dependent on the timing and duration of exposure (87). We found a shift of sex ratios towards females for both the 0-90 dpf and 60-90 dpf exposures to EE2, although these were less pronounced for the exposure in later life (6:7 and 1:11 male:female respectively; in controls there was a 2:1 male bias), suggesting a lower sensitivity of the gonads to this estrogenic effect after 60 dpf. The observed bias towards females in the exposure to 100% effluent may have been influenced by the additional nutritional sources in the effluent; in fish an enhanced growth rate during the critical period of gonadal differentiation is thought to be either a trigger of, or a key influence on, ovarian differentiation (88). That growth plays a key role in sex determination in fish is consistent with other reports of higher proportions of females amongst the largest fish found within wild populations of fish more generally (89, 90).

We found no evidence of intersex fish (fish containing both oocytes and male germ cells in the gonad) in either 50% or 100% effluent or the EE2 exposure at any of the measured developmental time-points. In other fish species intersex is commonly associated with exposure to steroidal estrogens and has been widely documented in fish following exposure to EE2 and WwTW effluents (2, 4, 91–93). However, our finding supports those from Örn et al. (70), who showed that whilst exposure to 10 ng/L EE2 induced intersex in medaka (10% of the population) it did not do so in zebrafish. Gonadal sex differentiation in zebrafish is complex and still not understood fully; many males, but not all (55, 56) have juvenile ovaries prior to differentiation into mature testes (94, 95).

Interestingly, effects of exposure to 100% effluent or EE2 on gonadal development for both the longer and shorter exposure time windows showed contrasting sex dependent outcomes. In males there was a delay in testis maturation, whereas in females, ovary development was accelerated (as shown by others for EE2 (55, 56, 81, 82, 96). Such contrasting effects for exposure to estrogenic WwWT effluents could therefore de-synchronize readiness for breeding between the sexes.

### Tissue GFP Protein Expression

One of the major advantages of estrogen responsive biosensor transgenic fish is the ability to visually identify tissue-specific responses to estrogen. In EE2 exposed fish, the tissue responses at 4 dpf in liver, heart, forebrain, otic vesicle and somites are consistent with those observed previously (37, 38), with similar responses also at 21 dpf and 30 dpf. GFP expression in the skeletal tissue at 30 dpf however indicated wider potential estrogen targets at this life stage. Several studies have now suggested that various environmental estrogens can impact on skeletal development and morphology in fish (97–99).

In the 0-90 dpf exposure to 100% WwTW effluent, GFP expression occurred in the heart, otic vesicle and somites only, but again the tissues that were responsive varied for the different developmental stages (e.g., at 30 dpf, GFP expression was observed in the somites only). These differences may relate to differences in the ontogeny of expression of the estrogen receptors in the different tissues and/or differences in the chemical mix in the effluent; for example, the presence of chemicals that might inhibit the estrogen responsive pathway. The lack of any GFP induction in 30 dpf fish exposed to 100% effluent during the period of sexual differentiation (21-60 dpf) likely relates to a combination of the relatively short duration of exposure to this time point and low levels of the three steroidal estrogens at this time.

### *vtg* and *gfp* mRNA Induction

Induction of *vtg1* mRNA occurred in very early life stages, consistent with other studies (68, 100, 101) and there was a progressive increase in *vtg1* mRNA over time in fish chronically exposed to both 100% effluent and EE2, which may suggest an accumulation of environmental estrogen(s) in the fish over time. Comparing the responses of *vtg* mRNA with *gfp* mRNA for the chronic exposure (0-90 dpf) and across the different developmental stages, we show that both biomarkers responded to environmentally relevant concentrations of estrogen(s), albeit the dynamic range of the response for the induction *vtg* was far greater when compared with *gfp*.

Interestingly, correlations between the expression levels of *vtg* and *gfp* mRNA for both the 100% effluent and EE2 chronic (90 day) exposures were weak at 21 dpf (whole bodies; r^2^ = 0.2) which may indicate a lower responsiveness of the *vtg1* gene to estrogen stimulation prior to gonadal sexual differentiation. From this time onwards in the chronic exposure (0-90 dpf), and for exposures for the life periods 21-30 dpf, 30-60 dpf and 60-90 dpf, the correlations were strong for EE2 and moderate for the WwTW effluent. The overall weaker correlations for the WwTW effluent compared with that for EE2 may reflect differences in the induction for *vtg1* versus *gfp* mRNA for the complex mixtures of chemicals contained in the WwTW effluent. The overall stronger positive correlations for induction for *vtg1* versus *gfp* mRNA for the liver tissues compared with those for whole body homogenates (early life stages) are likely due to the fact that *vtg1*mRNA is exclusive to the liver, whereas *gfp* mRNA was produced across multiple tissues which varies across developmental stages.

## Conclusion

We show that health impacts for exposure to environmental estrogens in zebrafish varies with duration and the life stage exposed. We further illustrate the utility of the transgenic ERE-GFP-Casper zebrafish model as an experimental model for building understanding and exploring effect mechanisms for exposure to estrogens and their environmental mixtures and informing on their adverse outcome pathways.

## Data Availability Statement

The raw data supporting the conclusions of this article will be made available by the authors, without undue reservation.

## Ethics Statement

The animal study was reviewed and approved by conducting experimental procedures under a UK Home Office (Scientific Procedures) Animals Project License (PPL30/3430) and under strict adherence to local guidelines at the University of Exeter, overseen by an Ethical Review Board (with membership that includes independent authorities and members of the lay public).

## Author Contributions

The project was conceived and designed by CRT and RC. RC carried out the exposure studies and data analysis. AL provided general project support and expert guidance on the molecular and histological analysis. AD carried out the analytical chemistry. RC and CRT wrote the manuscript, with input from AL and AD. All authors contributed to the article and approved the submitted version.

## Funding

RC was funded by a Biology and Biotechnology Research Council studentship (BB/K501281/1). The project was further supported by the University of Exeter on grants to CRT. AD was supported by a Marie Curie Intra European Fellowship within the European Community Seventh Framework Programme ([FP7/2007-2013]) under grant agreement no.: 302097.

## Conflict of Interest

The authors declare that the research was conducted in the absence of any commercial or financial relationships that could be construed as a potential conflict of interest.

## Abbreviations

ANOVA, analysis of variance; BOD, biochemical oxygen demand; BPA, bisphenol-A; dpf, days post fertilization; E1, estrone; E2, 17β-estradiol; EDC, endocrine disrupting chemical; EE2, 17α-ethinylestradiol; ERE, estrogen responsive element; GFP, green fluorescent protein; hpf, hours post fertilization; NP, 4-nonylphenol; PE, population equivalent; RT-qPCR, real-time quantitative polymerase chain reaction; SPE, solid phase extraction; SWC, system water control; TSS, total suspended solids; UHPLC-MS/MS -ultra high-performance liquid chromatography tandem mass spectrometry; VTG – vitellogenin; WwTW, wastewater treatment work.
