# Supplementary material for: Health Effects and Life Stage Sensitivities in Zebrafish Exposed to an Estrogenic Wastewater Treatment Works Effluent
Source: Front Endocrinol (Lausanne). 2021 Apr 30;12:666656. doi: 10.3389/fendo.2021.666656 (PMC8120895; doi:10.3389/fendo.2021.666656)
Supplement: Supplementary file 1 [file DataSheet_1.pdf]

Health effects and life stage sensitivities in zebrafish exposed to an estrogenic wastewater treatment works effluent

Ruth Cooper[1], Arthur David[2‡], Anke Lange[1], Charles R. Tyler[1*]

[1] University of Exeter, Biosciences, College of Life & Environmental Sciences, Exeter EX4 4QD, United Kingdom

[2] University of Sussex, School of Life Sciences, Brighton BN1 9QJ, United Kingdom

*** Correspondence:**
University of Exeter, Biosciences, College of Life & Environmental Sciences, Stocker Road, Exeter, Devon, EX4 4QD, UK
Tel: +44 1392 264450
E-mail: C.R.Tyler@exeter.ac.uk

[‡] **Present Address:**
Univ Rennes, Inserm, EHESP, Irset (Institut de recherche en santé, environnement et travail) – UMR_S 1085, F-35000 Rennes, France

Supplementary Method	Protocol for SPE and LC-MS…………………………..	Page S2
Supplementary Method	Protocol for gonadal histopathology…………………...	Page S3
Table S1	Retention times and MRM conditions used for UHPLC–ESI-MS/MS analysis of steroidal estrogens…………....	Page S4
Table S2	Information on the study WwTW……………………...	Page S5
Table S3	Measured concentrations of environmental estrogens for the different consignments of effluent collected……….	Page S6
Table S4	Developmental staging system for the testis and ovary..	Page S7
Table S5	Overview of RT-qPCR parameters…………………….	Page S8
Figure S1	Diagrammatic representation of the exposure system….	Page S9
Figure S2	Staging of gonadal development……………………….	Page S10
Figure S3	Correlation between *gfp* and *vtg* mRNA expression in ERE-GFP Casper zebrafish…………………………….	Page S11
Supplementary References	……………………………………………………...	Page S12

Supplementary Methods

SPE protocol and quantification of steroidal estrogens in the WwTW effluent using LC-MS

Clean up using DSC-NH2 cartridges was conducted according to Flores et al. (1). Briefly, 0.25 mL of the dried Oasis HLB extracts were first dissolved in 1 mL of ethyl acetate. DSC-NH2 cartridges were then conditioned with 1 mL of ethyl acetate and 1 mL of ethyl acetate/methanol (4:1, v/v). The extract was loaded onto the cartridge and the eluate was directly collected. The cartridges were further rinsed with 2 mL of the solvent mixture and the final extracts were taken to dryness. After this, extracts were reconstituted in 100 µL of water/acetonitrile (7:3, v/v) and passed through 0.22 µm centrifuge filters and stored at -80 °C until analyses.

Ultra high-performance liquid chromatography tandem mass spectrometry (UHPLC-MS/MS) analyses was carried out using a Waters Acquity UHPLC system coupled to a Quattro Premier triple quadrupole mass spectrometer (Micromass;Waters, Manchester, UK). Samples were separated using a reverse phase Acquity UHPLC BEH C18 column (1.7 µm particle size, 2.1 mm × 100 mm, Waters, Manchester, UK) fitted with an Acquity UHPLC BEH C18 VanGuard pre-column (130 Å, 1.7 µm, 2.1 mm x 5 mm, Waters, Manchester, UK) maintained at 25 °C. The injection volume was 20 µL and the mobile phase solvents were 94.95% water, 5% MeOH, 0.05% ammonium hydroxyde (A) and 99.95% MeOH, 0.05% ammonium hydroxyde in an initial ratio (A:B) of 40:60. Separation was achieved using a flow rate of 0.15 mL/min with the following gradient: 40:60 to 20:80 over 10 min; then from 20:80 to 0:100 and held for 5 min, then with a return to initial condition at 15 min and with equilibration for 7 min. Retention times, ionisation and fragmentation settings are reported in Table S1. MS/MS was performed in the Multiple Reaction Mode (MRM) using ESI in the negative mode and two characteristic fragmentations of the deprotonated molecular ion $[M{-}H]^{-}$ were monitored. The first and most abundant one was used for quantitation, while the second was used as a qualifier. Other parameters were optimised as follows: capillary voltage −3.13 kV, extractor voltage 8 V, multiplier voltage 650 V, source temperature 120 ◦C, desolvation temperature 400 ◦C. Argon was used as collision gas (P collision cell: 3×10^{-3} mbar), and nitrogen was used as both the nebulizing (100 L/h) and desolvation gas (600 L/h). Mass calibration of the spectrometer was performed with sodium iodide. Data were acquired using MassLynx 4.1 and the quantification was carried out by calculating the response factor of estrogenic compounds to internal standards. Concentrations were determined using a least-square linear regression analysis of the peak area ratio versus the concentration ratio native to

deuterated. At least, five-point calibration curve ($R2 > 0.99$) covering the range of concentrations for all compounds were used within the linear range of the instrument.

Protocol for gonadal histopathology

All fish samples were fixed *in toto* in Bouin's fixative (Fisher Scientific) for a maximum of 6 hours and washed twice in 70% ethanol (at 12 and 24 hours post sampling) and subsequently processed for 16 hours in a semi-enclosed tissue processor (Shandon Citadel 2000). In brief, tissue samples were progressively dehydrated through a series of graded industrial methylated spirits (IMS) solutions (70-100%), washed in 100% ethanol and Histoclear and then embedded in paraffin wax at 60 °C. A minimum of 30 zebrafish per treatment were then serially sectioned into 5 µm sections on a rotary microtome (Leica model). For each fish, a minimum of 6 transverse serial sections were obtained (taken 50 µm and 250 µm apart along the gonad for males and females, respectively) mounted onto glass slides and subsequently stained with hematoxylin- eosin, before examination using an Axioscope 40 light microscope (Zeiss, Oberkochen, Germany) at magnifications between 10-40x to determine both gonadal sex and maturity (see Supplementary Figure S2) and to assess for any alterations in the structural organization of the gonad, i.e. intersex. The stage of gonadal maturity was recorded according to the standardised criteria described in the OECD Histopathology Guidance document (2) and using a numerical staging system based on Johnson et al. (2) (see Supplementary Table S4).

Supplementary Table S1. Retention times and MRM conditions used for UHPLC–ESI-MS/MS analysis of 17α-ethinylestradiol (EE2), 17β-estradiol (E2) and estrone (E1) in negative mode.

	**Retention time**	**MRM transitions**			**TQ parameters**	
Compound		Precursor	quantification ion	qualification ion	Cone (V)	Collision (V)
EE2	5.01	295	145	159	50	40
E2	5.08	271	145	183	50	45
E1	5.06	269	145	143	50	45
EE2-d4	4.97	299	147	161	50	50
E2-d4	5.04	275	147	187	50	50
E1-d4	5.04	273	145	147	50	45

Supplementary Table S2. Information about the WwTW the study effluent was collected from. Summary of treatment process type, including total hydraulic retention times (HRT) and sludge retention times (SRT), total population equivalent (P.E.) values and types of industrial discharges for the studied treatment works.

**Study WwTW**	
Total population equivalent	141698
Industrial population equivalent	4713
Proportion of industrial wastewater (%)	3.30
Secondary treatment	Activated sludge
Tertiary treatment	Nitrifying BAFF
	UV Disinfection
Total HRT (hrs)	6 AS/12BF
Total SRT (days)	6
Number of permitted industrial discharges	49
Types of industrial/trade waste	Car washes
	Plant wash down
	Laundrettes
	Rendering plant
	Electroplater
	University
	Cattle market
	Gas holders
	Swimming pools
	Food processing

BAFF = Biological aerated flooded filter
AS = Activated sludge
BF = Biological filters
HRT = Hydraulic retention time
SRT = Sludge retention time

The UK WwTW studied received waste primarily from domestic sources, with some industrial/trade waste. The total P.E. or unit per capita loading for this treatment works was 141698. P.E. This is a value for quantifying the organic strength of wastewater discharges and reflecting the net waste of the surrounding human population and industrial facilities and is based on a biological oxygen demand (BOD) of the water of 60g per person/per day. P.E. has been used also to predict/model steroid estrogen content within WwTWs. The total and industrial P.E. for the study WwTW was obtained from the water utilities responsible for its operation.

Supplementary Table S3. Measured concentrations of environmental estrogens for the different consignments of effluent collected during the experiment. Estrone (E1), 17β-estradiol (E2) and 17α-ethinylestradiol (EE2) were measured by UHPLC-MS/MS.

		**Measured concentration (ng/L)**								
		Consignment 1	Consignment 2	Consignment 3	Consignment 4	Consignment 5	Consignment 6	Consignment 7	Consignment 8	Consignment 9
**June**	Estrone (E1)	<MDL	<MDL	<MDL	<MQL	<MQL	4.8			
	Estradiol (E2)	<MQL	<MDL	<MDL	<MDL	<MDL	<MDL			
	Ethinylestradiol (EE2)	<MDL	<MDL	<MDL	<MDL	<MDL	<MDL			
**July**	Estrone (E1)	<MQL	<MDL	<MDL	<MDL	<MDL	<MQL	<MDL	<MQL	<MDL
	Estradiol (E2)	<MDL	<MDL	<MQL	<MDL	<MDL	<MQL	<MQL	<MDL	<MDL
	Ethinylestradiol (EE2)	<MDL	<MDL	<MDL	<MDL	<MDL	<MDL	<MDL	<MDL	<MDL
**August**	Estrone (E1)	<MQL	<MQL	<MDL	2.8	<MDL	4.0	4.1	4.1	
	Estradiol (E2)	<MDL	<MDL	<MQL	<MQL	<MQL	<MQL		<MQL	
	Ethinylestradiol (EE2)	<MDL	<MDL	<MDL	<MDL	<MDL	<MDL	<MDL	<MDL	
**September**	Estrone (E1)	2.7	7.2	3.4	39.6	35.2	24.7	25.8		
	Estradiol (E2)	<MQL	3.9	<MQL		46.4		12.9		
	Ethinylestradiol (EE2)	<MDL	<MDL	<MDL	<MDL	<MDL	<MDL	<MDL		

Method detection limits (MDL) were 0.2 ng/L (E1), 0.4 ng/L (E2) and 0.5 ng/L (EE2); Method quantification limits (MQL) were 0.6 ng/L (E1), 1.2 ng/L (E2) and 1.5 ng/L (EE2)

Supplementary Table S4: Developmental staging system for the testis and ovary.

**Ovary**	**Stages 0-5**
Stage 0	Undeveloped - entirely immature phases (oogonia to perinucleolar oocytes); no cortical alveoli
Stage 1	Early spermatogenic - vast majority (e.g., > 90%) are pre-vitellogenic follicles, predominantly perinucleolar through cortical alveolar.
Stage 2	Mid-development - at least half of observed follicles are early and mid-vitellogenic.
Stage 3	Late development - majority of developing follicles are late vitellogenic.
Stage 4	Late development/hydrated - majority of follicles are late vitellogenic and mature/spawning follicles; follicles are larger as compared to Stage 3.
Stage 5	Post-ovulatory - predominately spent follicles, remnants of theca externa and granulosa.
**Testes**	**Stages 0-4**
Stage 0	Undeveloped - entirely immature phases (spermatogonia to spermatids) with no spermatozoa.
Stage 1	Early spermatogenic - immature phases predominate, but spermatozoa may also be observed; the germinal epithelium is thinner than it is during Stage 2.
Stage 2	Mid-spermatogenic - spermatocytes, spermatids, and spermatozoa are present in roughly equal proportions; the germinal epithelium is thinner than Stage 1, but thicker than Stage 3.
Stage 3	Late spermatogenic - all stages may be observed, however, mature sperm pre-dominate; the germinal epithelium is thinner than it is during Stage 2.
Stage 4	Spent - loose connective tissue with some remnant sperm.

The staging criteria used were based on the OECD Histopathology Guidance document (2).

Supplementary Table S5: Overview of RT-qPCR parameters

**Gene name**	**Gene symbol**	**Forward primer (5'-3')**	**Reverse primer (5'-3')**	**Ta (°C)**	**Efficiency (%)**	**Reference [a]**
Vitellogenin 1	*vtg1*	GCCAAGAAAGAACCCAAACTG	GGAGATGAGAGCCACTGAAG	62.0	110	(3)
Ribosomal protein L8	*rpl8*	CCGAGACCAAGAAATCCAGAG	CCAGCAACAACACCAACAAC	59.5	102	(4)
Enhanced green fluorescent protein	*egfp*	CGACGGCAACTACAAGAC	TAGTTGTACTCCAGCTTGTGC	60.0	96.5	n/a

[a] Primer sequences were obtained from these references, but assays were optimized as described; n/a: these primers were designed for this study.

Supplementary Figure S1. Diagrammatic representation of the exposure system used for delivering the effluent from the storage tank to the exposure tanks.

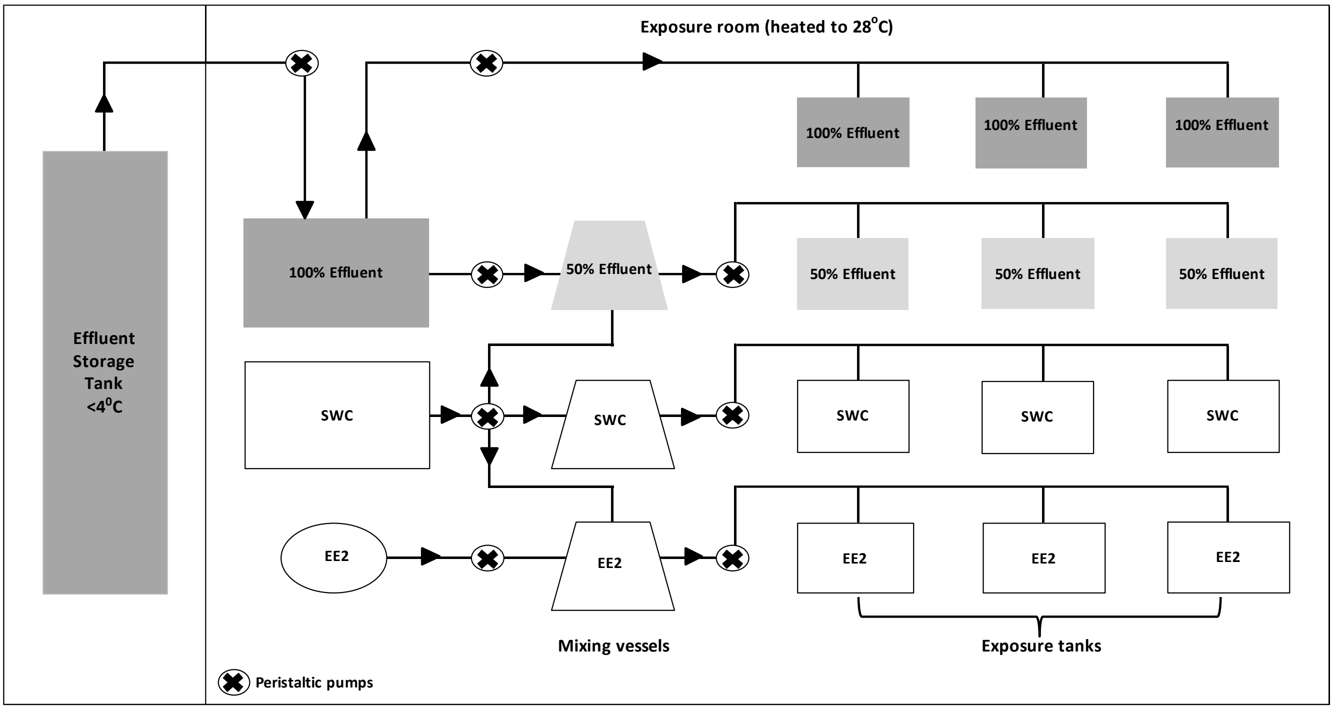

A series of peristaltic pumps were used to deliver the effluent, dilution water and estrogen positive control (EE2) to the respective mixing vessels and then from each mixing vessel to the replicate exposure tanks for each graded effluent treatment (50 and 100% effluent), the system water control (SWC) and the positive control (EE2). The treatments were randomly distributed within the exposure room but are shown here ordered for ease of presentation.

Supplementary Figure S2. Staging of gonadal development.

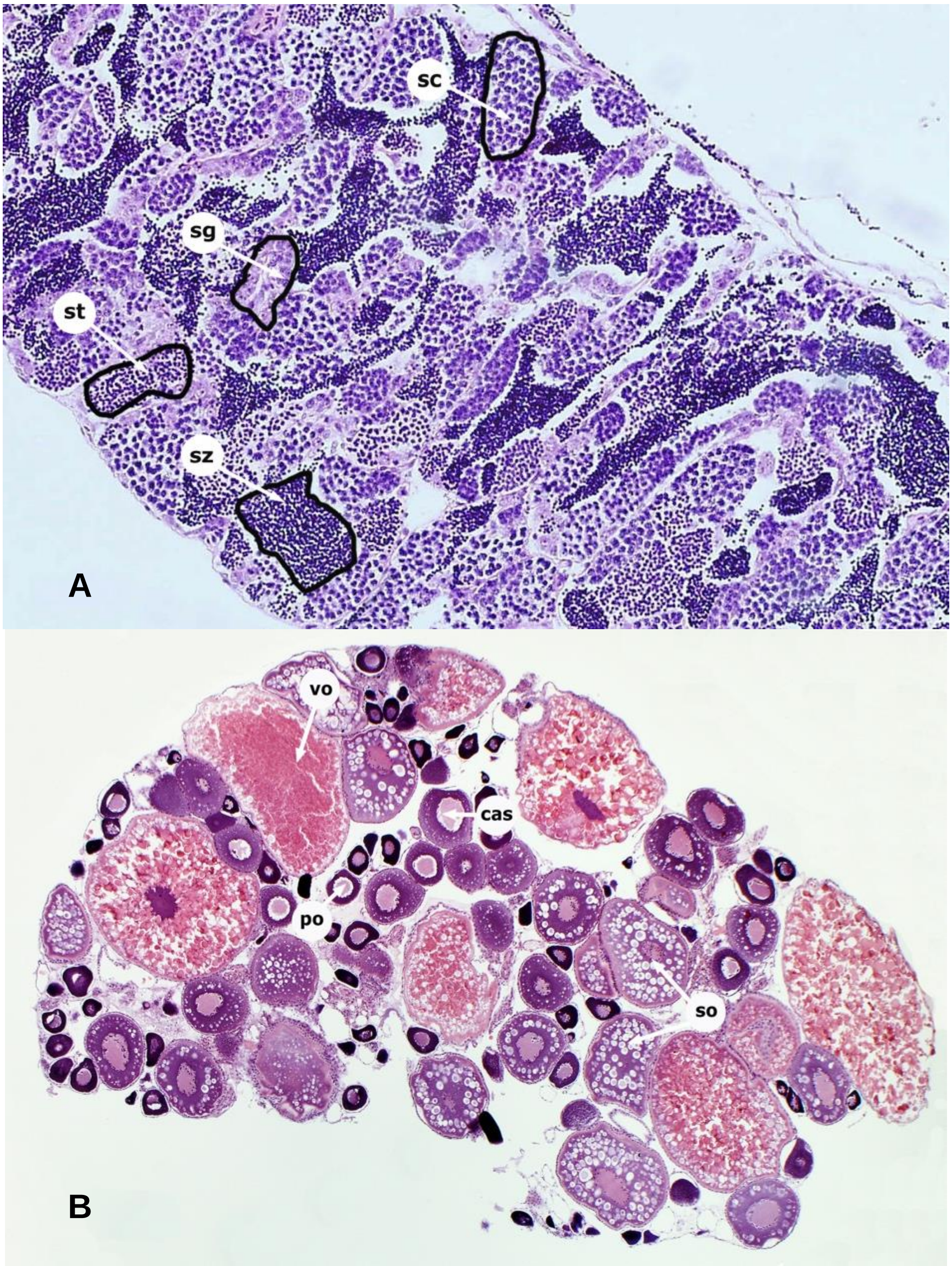

Histological sections of a testis (a) and ovary (b)showing the different germ cell types observed during the spermatogenesis and oogenesis, respectively in ERE-TG Casper zebrafish. The stage of maturity of the gonad was assigned based on OECD guidelines by means of a numerical system i.e., ovary: stages 0 - 5, testis: stages 0 - 4 (see Table S3). The order of least to most advanced germs cells during spermatogenesis is: spermatogonia (sg), spermatocytes (sc), spermatozoa (sz) and spermatids (st). The order of least to most advanced germs cells during oogenesis is: primary oocyte (po), cortical alveolous stage (cas), secondary oocyte (so) and vitellogenic oocyte (vo).

Supplementary Figure S3. Correlation between *gfp* and *vtg* mRNA expression in ERE-GFP Casper zebrafish.

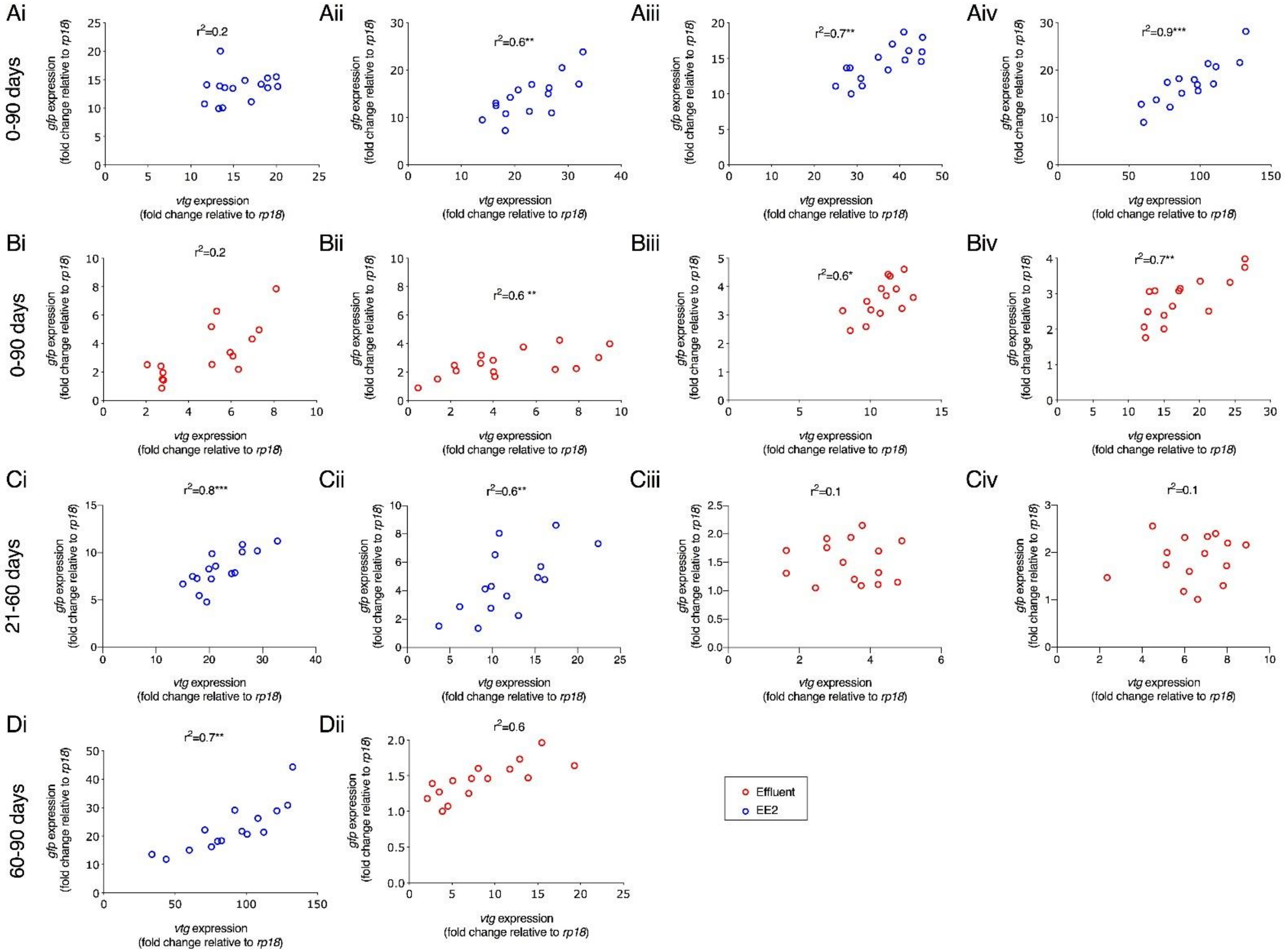

Transgenic zebrafish were exposed to EE2 (10 ng/L) or a graded effluent (50% and 100%) and *gfp/vtg* expression measured at the following life stages: 21 dpf (Ai;Bi), 30 dpf (Aii;Bii;Ci), 60 dpf (Aiii;Biii;Cii) and 90 pdf (Aiv;Biv; Dii). Data are reported as mean ± SE and expressed as fold-change compared with the control.

Supplementary References

1. Flores A, Hill EM. Formation of estrogenic brominated ethinylestradiol in drinking water: Implications for aquatic toxicity testing. *Chemosphere* (2008) **73**:1115–1120. doi:10.1016/j.chemosphere.2008.07.022

2. Johnson R, Wolf J, Braunbeck T. OECD Guidance document for the diagnosis of endocrine-related histopathology of fish gonads. (2009).

3. Lange A, Katsu Y, Miyagawa S, Ogino Y, Urushitani H, Kobayashi T, Hirai T, Shears JA, Nagae M, Yamamoto J, et al. Comparative responsiveness to natural and synthetic estrogens of fish species commonly used in the laboratory and field monitoring. *Aquatic Toxicology* (2012) **109**:250–258. doi:10.1016/j.aquatox.2011.09.004

4. Santos EM, Workman VL, Paull GC, Filby AL, Van Look KJW, Kille P, Tyler CR. Molecular basis of sex and reproductive status in breeding zebrafish. *Physiological Genomics* (2007) **30**:111–122. doi:10.1152/physiolgenomics.00284.2006
